# Practical guide to quantification of hepatic iron with MRI

**DOI:** 10.1007/s00330-019-06380-9

**Published:** 2019-08-07

**Authors:** Benjamin Henninger, Jose Alustiza, Maciej Garbowski, Yves Gandon

**Affiliations:** 1 Department of Radiology, Medical University of Innsbruck, Anichstraße 35, 6020 Innsbruck, Austria; 2 Osatek, Donostia Universitary Hospital, P. Dr. Beguiristain 109, 20014 Donostia/San Sebastian, Spain; 3 Department of Haematology, Cancer Institute, University College London, Paul O’Gorman Bld, 72 Huntley St, London, WC1E 6BT UK; 4 CHU Rennes, Inserm, LTSI - UMR_S 1099, University of Rennes, F-35000 Rennes, France

**Keywords:** Iron, Liver, Magnetic resonance imaging

## Abstract

**Abstract:**

Our intention is to demystify the MR quantification of hepatic iron (i.e., the liver iron concentration) and give you a step-by-step approach by answering the most pertinent questions. The following article should be more of a manual or guide for every radiologist than a classic review article, which just summarizes the literature. Furthermore, we provide important background information for professional communication with clinicians. The information regarding the physical background is reduced to a minimum. After reading this article, you should be able to perform adequate MR measurements of the LIC with 1.5-T or 3.0-T scanners.

**Key Points:**

• *MRI is widely accepted as the primary approach to non-invasively determine liver iron concentration (LIC).*

• *This article is a guide for every radiologist to perform adequate MR measurements of the LIC.*

• When using R2* relaxometry, some points have to be considered to obtain correct measurements—all explained in this article.

## Introduction and overview

Magnetic resonance imaging (MRI) is widely recognized as the primary approach to non-invasively determine liver iron concentration (LIC). Over the past 20 years, various methods have been extensively studied and eventually introduced into routine clinical management in many centers. Nevertheless, in our experience, it seems that many radiologists are still “afraid” of this method and therefore do not use it or use it inappropriately. The likely reason for this seems to be its apparently complex background and the many different approaches on offer. As a result, although the benefits of MRI in the diagnosis and management of iron overload are at hand, MRI has only been included in a few clinical guidelines and recommendations [1–6] and is therefore actually not seen as a mandatory method that ought to be offered by every radiologist working with MRI.

## Which MRI techniques are available?

**Fig. 1:**
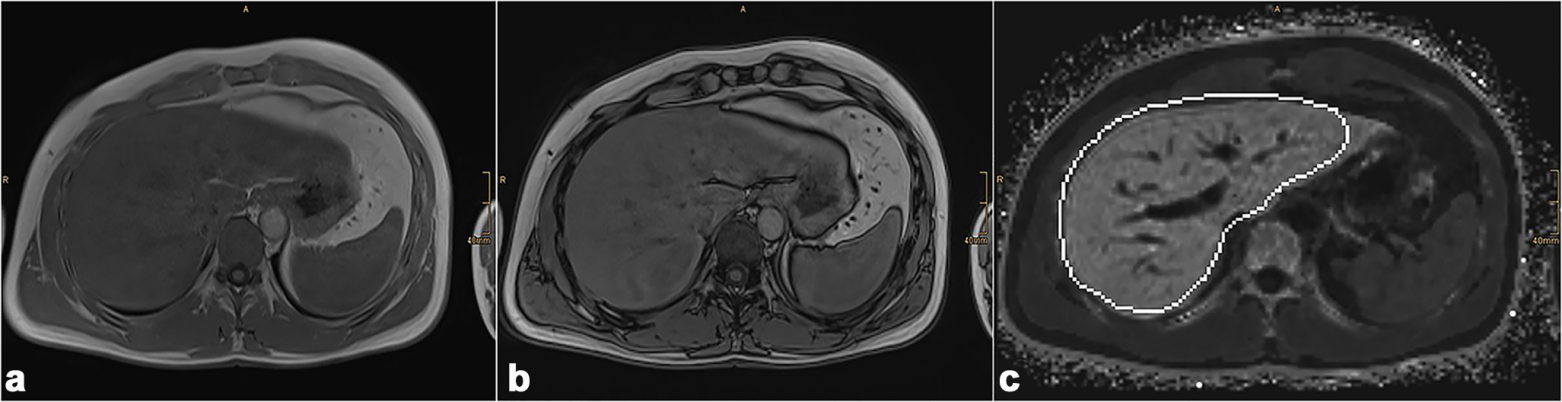
Example of a chemical shift sequence which already indicates a pathological iron deposition. The signal intensity of the liver in in-phase (**a**, TE = 4.77 ms, TR = 6.68 ms) is decreased (SI 100) compared to out-phase ((**b**) SI 120, TE = 2.35, TR = 6.68 ms) suggesting iron overload. Multi-echo gradient-echo sequence (**c**) provides a R2* of 128 s^−1^, which corresponds to a pathological LIC of ~ 67 μmol/g

There is an easy way to get an idea or first impression of a possible iron overload in the liver with MRI: nearly every MRI protocol of the liver integrates a chemical shift sequence, i.e., in- and opposed-phase. Iron leads to decreased signal intensity on in-phase images compared with the opposed-phase which in turn should alert on a possible iron overload disease [7, 8]. An example is provided in Fig. 1. A quantification method has been proposed by Lim [7]. However, concomitant steatosis is a crucial limitation of this technique which compromises its value [9, 10]. Further, fast spin-echo T2-weighted imaging can also be used to detect iron: the T2 shortening leads to low liver signal intensity, relative to that of the spleen.

There are two more advanced methods that can determine the LIC quantitatively: relaxometry and the signal-intensity-ratio method.

### Relaxometry

Relaxometry is the quantitative evaluation of the MRI signal loss due to the predominant shortening of the T2 and moreover the T2* relaxation times. There are two approaches: the calculation of the T2 time constant, based on spin-echo sequences, and of the T2* time constants, based on gradient-echo sequences. Both can be estimated from signal intensity decay acquired at multiple echo times (TEs). Because of the increase in the presence of tissue iron and therefore more logical applicability, we use the mathematical inverses of T2 and T2*, the R2 and R2* relaxation rates, for daily routine and regulatory purposes in the liver.

R2 Relaxometry - Ferriscan*®* (St. Pierre’s method) is a commercially available and FDA-approved technique for 1.5-T scanners, based on five T2-weighted spin-echo (SE) acquisitions during free-breathing with increasing TEs for the calculation of R2 [11]. Its advantages are undeniable showing excellent correlation with the LIC and it is used in many clinics worldwide as well as in various studies, often determined as the “gold-standard” due to its cross-site and cross-platform validation and an ongoing data quality control/assurance. Nevertheless, this technique requires long imaging time (~ 20 min), with the definitive need for sedation in pediatric imaging, complex data processing with centralized data analysis (takes 2 business days to return a report), and a former calibration of instruments. Furthermore, a corresponding service fee per patient is charged with this method for the data analysis. On top of the costs of the MRI scan itself, this narrows its widespread adoption.

**Fig. 2:**
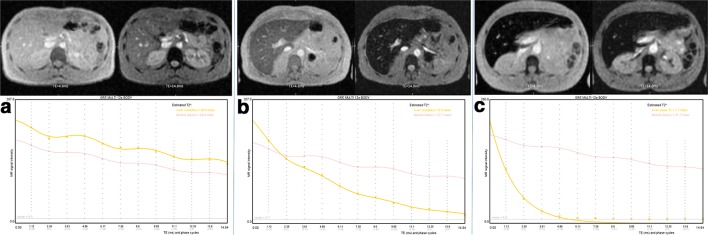
Three examples (**a**–**c**) of a liver ME-GRE sequence obtained at 1.5 T with 12 echoes showing for each patient a selection of TE = 4.8 and 14.8 ms images (first line) and also a MRQuantif graph (second line) plotting the signal intensity according to TEs (signal of liver is yellow, signal of muscle in light red). **a** Patient without iron overload (LIC 12 μmol/g). Visually, the liver signal is close to that of the paraspinous muscles on both echoes. On the graph, curve of the liver signal (yellow line) decreased progressively but stayed above that of the muscle (light red line). The slight sinusoidal ripple of the signal according to the phase corresponded to a mild degree of fatty infiltration. **b** Mild iron overload (LIC 93 μmol/g). Visually, the liver signal is below that of the paraspinous muscles, particularly on the long TE. On the graph, the liver signal decreased more rapidly than that of the muscle. **c** Major iron overload (LIC 355 μmol/g). Visually, the liver signal is collapsed on both echoes. On the graph, the liver signal decreased very rapidly and reached the level of the background noise at the fourth echo (4.8 ms)

R2* relaxometry has emerged as a reliable method providing a linear correlation with the LIC. It has shown superb reproducibility but the fact that sequence parameters and image analysis procedures were different among many studies has always been portrayed as a disadvantage [12–15]. Although there is no actual consensus on the ideal image acquisition, many centers are using R2* relaxometry with their house-made sequence and post-processing software with own LIC calibration. Nevertheless, studies have shown that the existing biases are correctable and, in some cases, also negligible if some individual points are considered, providing clinically acceptable estimation of the LIC with reproducible results [14, 16–18]. R2* relaxometry has further emerged as a very quick technique, acquired in only one breath-hold. With a first TE about 1 ms, the quantification of the LIC is possible up to 20 mg/g dry weight with 1.5-T scanners [19]. Nevertheless, we have to be aware that there remains an inaccuracy in such high iron values. Examples for using relaxometry in different patients are provided in Fig. 2. One of the major advantages of R2* relaxometry is the possibility of 3D acquisitions and parallel imaging, which allow to acquire a complete volumetric coverage of the liver.

### Signal-intensity-ratio

**Fig. 3:**
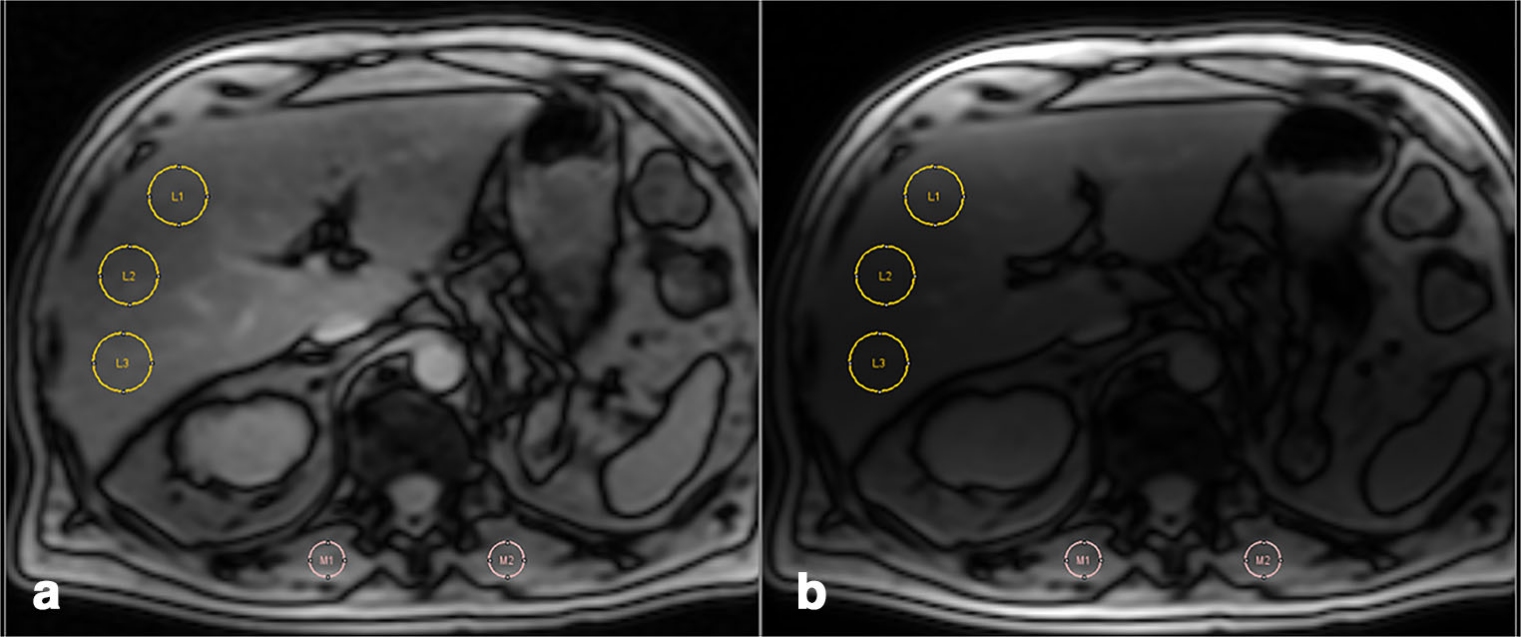
SIR method applied correctly on an image (**a**) acquired with the body coil providing a normal liver to muscle ratio of 0.97. The wrong application is shown in image (**b**) on the same patient acquired using surface coil with a liver to muscle ratio of 0.43. This may lead to a crucial mistake with the erroneous assumption that there is a significant iron overload. This is caused by the signal increase of the body parts closest to the surface coil

In 2004, Gandon and colleagues introduced the so-called signal-intensity-ratio (SIR) method (imagemed.univ-rennes1.fr) which is based on measuring the signal-intensity-ratio between the liver and the paraspinal muscles. It is performed by obtaining multiple breath-hold gradient-echo (GRE) sequence acquisition with 3 different TEs (4, 9, and 14 ms) and 20° flip angle, and the time to repetition (TR) is constant at 120 ms [20]. The model of the Spanish Society of Abdominal Imaging (SEDIA) proposed by Alustiza et al uses the same method with only 2 echoes (4 and 14 ms) and a different mathematical formula to calculate the LIC [21]. The results of the SEDIA’s model are better correlated with R2* and with LIC measured on liver biopsies [22]. In case of high iron overload, an add-in sequence using a shortest TE was proposed by Rose et al [21, 23]. With SIR, it is important to only use the body coil, and no surface coils should be selected (especially those integrated in the patient’s bed) to avoid any signal gradient between the surface and the depth (Fig. 3). This leads to a reduction of the signal-to-noise performance which ultimately limits the dynamic range of SIR and therefore induces bias (due to the use of magnitude images). Another limitation of SIR is that the technique does not correct for fat, despite the fact that it uses “in-phase” echoes [24]. This can also lead to a relevant bias.

## Which MRI technique is preferable?

For a long time, the R2* and SIR methods were opposed. They each have their limits.

The most significant advantages of SIR methods are their accessibility, being feasible on every machine in the world. With the improvement of ME-GRE sequences, SIR method is now considered less precise than R2* relaxometry for low or moderate iron overload. It can strongly overestimate the result if the measurements are made, by mistake, on images acquired with a surface coil.

The R2* method presents a risk for major underestimation of the overload if the signal is already collapsed at the first echo. This can be avoided at 1.5 T, in most cases, by setting a first very short echo, less than 1 ms, but this may be insufficient in case of massive overload or even less on a 3-T device. In this case, the use of the body coil makes it possible to compare with the muscle and to provide a ratio correcting the calculation error of the R2*. Indeed, the R2* and SIR methods can be carried out jointly from a single sequence if the body coil is selected, which is helpful in case of high overload and at 3 T [25].

Whenever the decision on the appropriate MRI method is made, keep the same method in case of patient follow-up. This is especially important for therapy monitoring as these methods (like R2 and R2*) should not be used interchangeably in the same patient [26, 27].

The following paragraphs are now based on R2* as the method of choice but you should keep in mind its limitations, at 3 T and in case of high overload.

## Which MR protocol do I need for R2*?

Multi-echo gradient-echo (ME-GRE) sequences are means of choice to determine the R2*. As was done in early work on the R2* calculation, an ME-GRE sequence can be replaced by several GRE sequences with a single TE variation, but with the condition of not recalibrating between acquisitions [12].

The first echo time (TE) is the key parameter and should be chosen as short as possible, i.e., 1 ms or less [16, 28–30]. Because the effect on the signal decrease is proportional to the magnetic field, at 3 T, to get the same level of result, the TE values should be divided by 2. This explains the limitation of quantifying high LIC at that field because it is difficult, at least routinely, to obtain the first TE below 0.5 ms. Furthermore, an appropriate number of echoes with short echo spacing (around 1 ms) should be used, and in literature, all calibrated sequences never had less than 8 echoes and we suggest at least 12 echoes. The TR is usually being set between 25 and 120 ms with a low flip angle, which is important if the same sequence is also used to quantify fat.

The use of fat saturation can lead to systematically lower R2* values [31, 32]. This effect is significant for R2* > 300 s^−1^ (when fat and water peaks begin to overlap) and therefore not relevant in patients with hereditary hemochromatosis (HH) or dysmetabolic iron overload syndrome (DIOS) with expected R2* values far below this threshold. It has been shown that the spectral complexity of the fat signal introduces errors in R2* quantification in the presence of high fat concentration [33]. Using multi-peak modeling can mitigate those effects on R2* measurements. Applying the fat saturation on the sequence used seems to be a possible solution to this; however, most of the existing R2*-LIC biopsy calibrations have been mainly derived in the absence of fat suppression [34]. Moreover, joint quantification of steatosis could be necessary in a context of metabolic syndrome. Therefore, we advise to not use fat suppression for routine work to simplify the approach.

A single transverse slice through the liver (at the same time through the spleen) is traditionally acquired in clinical practice, with only one breath-hold. All measurements should be performed prior to any administration of intravenous gadolinium chelate because this can change the clinically relevant results, especially with hepatocyte-specific contrast agents [35].

## How do I measure the R2*?

R2* is usually reported in sec^−1^ (s^−1^) in which T2* is simply its reciprocal (i.e., R2* = 1000/T2*, T2* is reported in ms). T2* can easily be converted by applying the factor of 1000, e.g., a T2* of 14 ms corresponds to 71.4 s^−1^ (1000/14 = 71.4). We suggest using R2* rather than T2* in your report, because R2* is directly proportional, rather than inversely, to iron.

An important part is the application of the fitting algorithm on the average signal intensity at various echo times. The following decay models are available: mono (single) exponentials with/without truncation or with constant or variable offset, complex or simple fitting, basic, baseline subtraction, subtraction of measured image noise; bi-exponential [36]. These data-fitting procedures need correcting for confounding effects, in particular image noise and signal modulations from fat. It is not yet clear which model appears to be the best, but the truncated model seems to be very accurate and the constant offset model very robust even for high iron levels [30]. The complex fit has been postulated as the best approach to avoid noise-related biases [37]. Nevertheless, as this is not a solution for daily clinical practice, we suggest that you adhere to two important aspects: decide on a decay model and stay with it, especially at follow-up examinations.

**Fig. 4:**
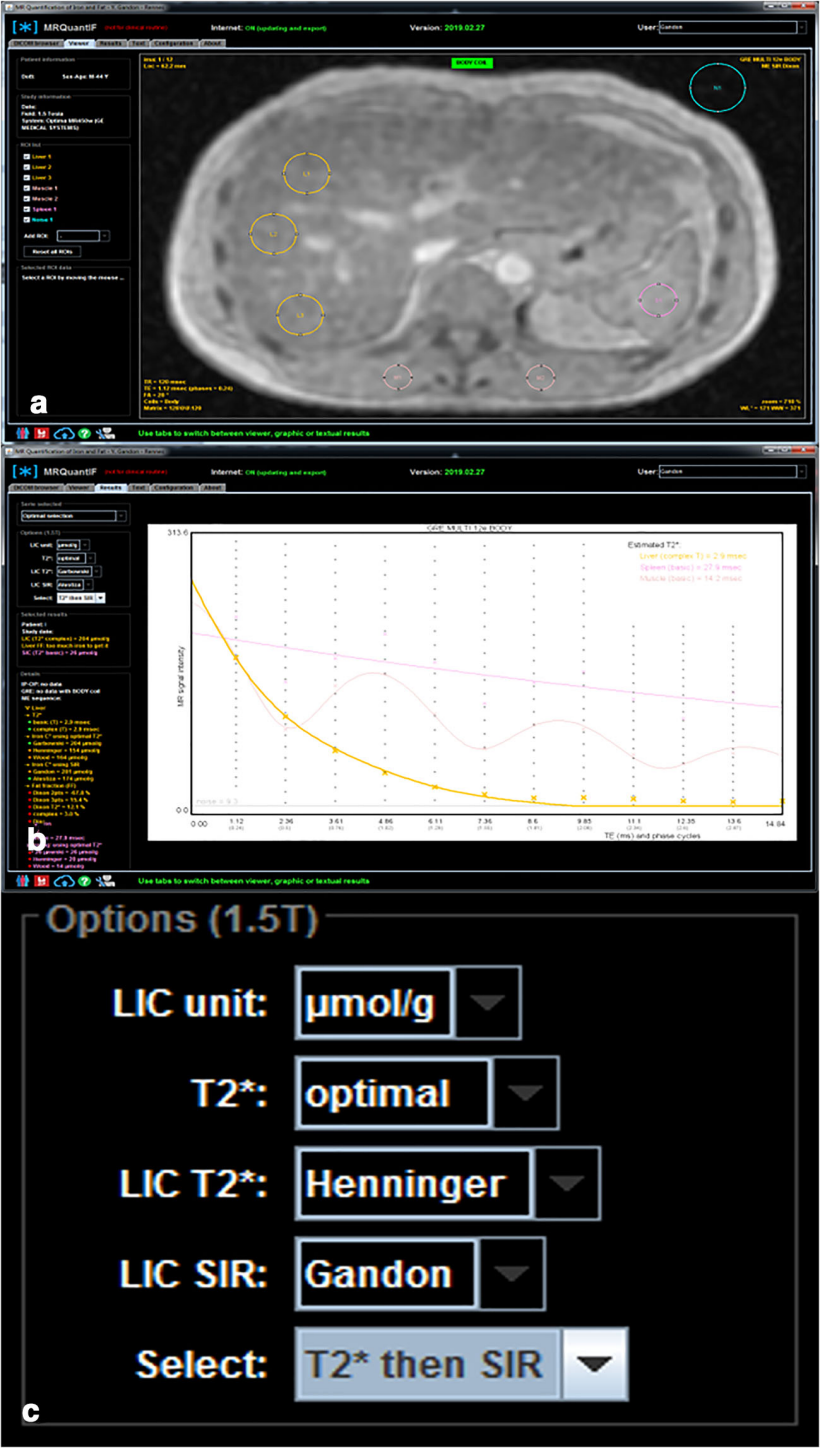
Example of a MR evaluation using the MRQuantif software. Selected ROIs are placed manually, three in the liver, one in the spleen, two in paraspinal muscles, and one in the background noise (**a**). The software then automatically calculates R2* (and T2*) values and further provides results calculated with the selected SIR method (**b**). The LIC is then stated for each method. It also allows the user to choose between different calibration formulas and fitting procedures (**c**)

Freely available software is available in which the group from Rennes recently launched, MRQuantif (http://mrquantif.org). This DICOM-dedicated solution automatically selects the best method and the preferable algorithm depending on the data provided but gives also the opportunity to select a fitting method and visualize the matching of curve to the data points (Fig. 4). This tool has the advantage to warn in case of incorrect or insufficient data. There are also other free tools building T2* maps, such as MRmap (https://sourceforge.net/projects/mrmap) or processing data allowing T2* calculation such as IronCalculator (http://www.ironcalculator.com) [38, 39]. We have to mention that all these free tools have no CE certification at the moment. The CMR tools (Cardiovascular Imaging Solutions, London, UK) provide a paid alternative. MRI vendors also propose dedicated tools like StarMap from General Electric or MapIt from Siemens Healthineers.

**Fig. 5:**
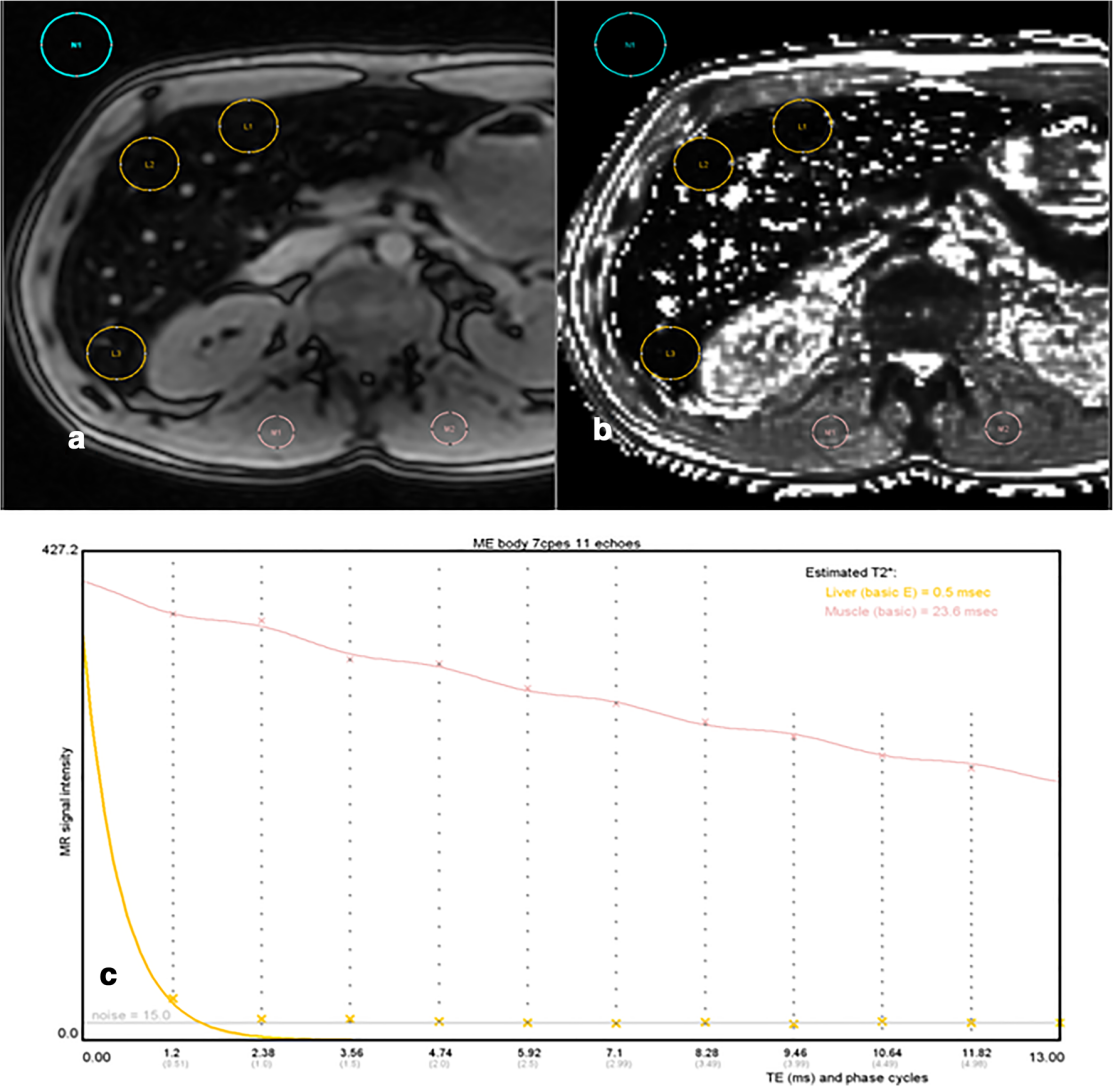
High LIC with 3-T imaging. **a** 2D ME-GRE sequence (first TE = 1.2 ms) obtained with body coil showing signal collapsed with a LIC of 521 μmol Fe/g as estimated by SIR method. In the same patient, pixelwise R2* map built by the 3D ME-GRE vendor solution (**b**), performed with surface coil, provides a wrong mean R2* of 188 s^−1^ which corresponds to slight iron overload (LIC = 59 μmol/g). The same patient was also scanned with another 3-T system from a different vendor (picture not provided) giving even a lower wrong R2* estimation by the 3D ME-GRE pixelwise map. R2* calculated using the same ROIs by MRQuantif (**c**) providing selected truncation fitting, excluding most of the points, was 1587s^−1^ corresponding to a LIC of 496 μmol Fe/g

MR vendors are proposing optional 2D or 3D multi-echo Dixon solutions integrating data processing, taking also fat influence into account. With General Electric, the product is called “IDEAL-IQ”, with Philips “StarQuant” (or mDixon-Quant), and with Siemens Healthineers “LiverLab” (or qDixon). They produce T2* or R2* but also fat fraction maps by doing a pixelwise fitting. An advantage that results from this is the possibility of calculating the proton density fat fraction. Some of these sequences have already been evaluated in literature with promising results at 1.5 T [40]. Comparison between 3D multi-echo Dixon approaches and the conventional, already-approved 2D GRE technique has shown excellent correlation [41]. Nevertheless, studies have shown some outliners or individual limitations such as reconstruction errors with fat-water swap. The first TE should be short enough to provide correct R2* evaluation (and consequently fat fraction) and to avoid a major LIC underestimation in case of high overload, particularly at 3 T (Fig. 5). We strongly recommend to first check that there is some liver signal left on the first two echoes of the native images before using the R2* map values.

The different approaches for placement of regions of interest (ROIs) or automatic whole-liver evaluation play a minor role in daily routine, and its pros and cons are negligible whereas whole-liver approaches are becoming favored in recent literature [42–45]. In general, several ROIs (2–3) should be drawn with 2–3 cm^2^, as large as possible [44]. Care should be taken to avoid large vessels or lesions [45]. Review the R2* images for iron heterogeneity and avoid measuring only in these areas due to the possibility of sampling errors.

## How to obtain the liver iron concentration from the R2*?

This is probably one of the most important steps. Again, the golden rule is to mention that you must stay with your method, especially when it comes to monitoring of therapy. In contrast to the MR-based determination of fat, the MR quantification of iron is not a direct method; it is based on calibration with biopsy. Therefore, we need a formula to translate the R2* into the LIC.

### 1.5 Tesla

The group by Wood et al was one of the first to report such a calibration-based formula (Fe in mg/g dry weight [R2*] = 0.0254 × R2* + 0.202) [12]. Other groups also performed such studies, and differences were as mentioned in sequence parameters as well as in post-processing [27, 46]. To transfer the R2* into the LIC, simply apply the measured R2* values to a calibration formula. Still, the question arises which calibration you should use? This depends on the sequence parameters you have chosen and then the applied fit. Next, choose the validated formula from Table 1 below. The LIC is reported either in mg Fe/g or μmol Fe/g dry liver tissue; a conversion of mg/g in μmol/g is done by a multiplication by the factor 18 (Fe[μmol/g] = 18 × Fe[mg/g]), i.e., in detail 1 μmol~55.845 μg (=atomic weight of iron).

**Table 1:** LIC calibration formulas at 1.5 T and R2* thresholds from literature

Study/method	Sequence; TR; TE; delta-TE; echoes; fit; fs	Calibration formulas to convert R2*[s^−1^] into LIC in mg/g and in μmol/g	Threshold for LIC > 2 mg/g or 36 μmol/g
Wood [12]	Single-echo gradient-echo; 25 ms; 0.8 ms; 0.25 ms; 16; variable offset; no fs	Fe [mg/g] = 0.0254 × R2* + 0.202 Fe [μmol/g] = R2* / 2.18 + 3.6	71 s^−1^
Garbowski [27]	Multi-echo gradient-echo; 200 ms 0.93 ms; 0.8 ms; 20; truncation; fs	Fe [mg/g] = 0.032 × R2* − 0.14 Fe [μmol/g] = R2* / 1.74–2.5	67 s^−1^
Henninger [16]	Multi-echo gradient-echo; 200 ms; 0.99 ms; 1.41 ms; 12; truncation; fs	Fe [mg/g] = 0.024 × R2* + 0.277 Fe [μmol/g] = R2* / 2.31 + 4.8	70 s^−1^
Hankins [46]	Multi-echo gradient-echo; not mentioned; 1.1 ms; 0.81 ms; 20; truncation; no fs	Fe [mg/g] = 0.028 × R2* × 0.454 Fe [μmol/g] = R2* / 1.98–8.1	88 s^−1^

*TR* repetition time, *TE* echo time, *fs* fat saturation, *LIC* liver iron concentration, *delta-TE* time between two echoes

The problem is that all available calibration formulas differ from each other, but why is this so? Firstly, all sequences have different acquisition parameters and post-processing fitting algorithms. Further differences in post-biopsy sample processing may also explain the difference between the calibration curves in literature [27]. These are the two major points. Nevertheless, it was shown that pooled data from studies that have a low initial TE in common provide relatively similar calibration results [16]. In general, when taking the studies by Wood, Garbowski, Hankins, and Henninger into account, a R2* threshold of 70 s^−1^ is in general a good surrogate and first orientation [12, 16, 27, 46]. Nevertheless, the studies by Kühn et al provide distinct different R2* thresholds [33, 47]. The reason can be seen in the histological evaluation of the liver biopsy samples (done by a subjective grading with no quantitative LIC determination), performing the MRI after the liver biopsy and the imaging parameters with a relatively low initial TE of 2.4 and only 3 acquired echoes, all based on a 3-point Dixon technique. Therefore, we must be aware when comparing different techniques and calibration with each other. Nevertheless, sequence parameters provided in Table 2 are a good orientation, rough deviations of which should be avoided.

**Table 2:** Sequence parameters for R2*

Sequence type	Gradient-echo sequence, breath-hold
Coil	At least at 3 T or in case of high overload use the body coil (coil autoselection should be switched off)
Plane/orientation	Axial
Field-of-view	38–40 cm
TR	~ 120 ms
TE initial	< 1 ms (as low as possible)
delta-TE	0.25–1.4 ms
Number of echoes	12–20
FA	20°
Options	No fat saturation is advised (depending on the calibration formula)
Slice thickness	7–10 mm
Number of slices	The maximum allowed, from the spleen to the pancreas

With the other abdominal organs (pancreas and spleen), there is no calibration that translates the R2* value into any quantitative unit for tissue iron but the same R2* technique as for the liver can be used. Pancreas and spleen R2* measurements can readily be obtained using the same approach as for liver R2*; they “come for free”. Schwenzer et al measured R2* of the liver, spleen, and pancreas in a healthy population, to get an idea of possible threshold [48]. They used a 12-echo gradient-echo sequence with fat saturation (first TE 2.6 ms), mono-exponential decay. The R2* range for the liver was 21.8–73.5 s^−1^ (*n* = 129 patients; R2* mean 35.6) which is also compatible with most studies. For the pancreas, the range was 15.4–38.6 s^−1^ (*n* = 61 patients; R2* mean 24.1) and for the spleen, 8.8–69 s^−1^ (*n* = 129 patients; R2* mean 22.8). A pancreas R2* of 100 s^−1^ appears to represent a risk threshold for predicting cardiac iron overload with a “clean” pancreas providing a nearly 100% negative predictive value for cardiac iron deposition [49]. For the spleen, 70 s^−1^ can be chosen as a reliable threshold, whereat 100 s^−1^ is a pathologic condition. R2* measurements of the pancreas and the spleen offer valuable information in the management of patients with hyperferritinemia [50].

### 3 Tesla

For the moment, there is only one reference analyzing the SIR and R2* methods in comparison with the LIC obtained by biopsy in a significant number of patients [25]. The conversion formula proposed from R2* calculated after subtraction of the background noise is LIC (μmol) = 0.314 R2* − 0.96. We could simplify in LIC (μmol) = R2* / 3.2. The increased sensitivity at 3 T allows for more precise analysis and detection of mild iron overload; conversely, it can be difficult to quantify severe iron overload beyond 150 μmol/g, unless the body coil is used to combine the two methods.

A complete checklist for the whole procedure is provided in Table 3.

**Table 3:** Checklist for LIC evaluation

SIR methods • You could use several single-echo GRE but preferably ME-GRE to reduce the acquisition time, to be able to combine both methods and to quantify fat. • For Rennes algorithm (for both 1.5-T and 3-T systems), use the protocol described on the https://imagemed.univ-rennes1.fr/en/mrquantif/protocols.php web page. For SEDIA protocol at 1.5 T, global parameters are identical but only the two echoes at 4 and 14 ms TEs are used. • Use only body coil! • To get LIC preferably, use the DICOM software MRQuantif to have a control of the coil selected or go on-line to mrquantif.org or www.sedia.es R2* methods • Use a 2D ME-GRE sequence (or several single-echo GRE if not available) and/or a vendor 3D ME-GRE optional sequence. • Check that the first echo is about 1 ms or even less. • Prefer a 2D ME-GRE sequence using body coil as described on the https://imagemed.univ-rennes1.fr/mrquantif/protocols if you are using a 3 T or dealing with highly overloaded patients • If you use a 2D ME-GRE sequence, choose your software option and the appropriate fit • Check that the R2* calculated is coherent with the liver signal • Select your R2* to LIC conversion formula • Mention the LIC and R2* values of spleen/pancreas in your report, define the thresholds for the clinicians	

## How to report?

The cut-off value for pathological LIC and hence iron overload has been defined as 36 μmol Fe/g or 2 mg Fe/g of dry weight [21]. The LIC should be calculated and reported; a simple R2* value of the liver does not help any clinician to get an idea of what is going on—calibration is the key [51]. It is important to speak one common language and the LIC is well known among clinicians. There is a terminology proposed in 2000 by EASL but a more detailed version taking in account more recent knowledge can be proposed (Table 4) [1].

**Table 4:** Proposition of a classification of iron overload severity

Limits			Iron overload	Comment
Upper limit (× normal)	μmol/g (approximately)	mg/g (approximately)		
< 1N	0 to < 36	0 to < 2	No	
< 2N	36 to < 75	2 to < 4	Insignificant	Usually with no treatment needed (except HH), follow-up
< 3N	75 to < 100	4 to < 6	Mild	Treatment depends on the patient profile
< 4N	100 to < 150	6 to < 8	Moderate	Treatment is usually performed (except hematologic causes)
< 8N	150 to < 300	8 to < 16	Moderate-severe	Corresponding usually only to HH or hematologic cause
≥ 8N	≥ 300	≥ 16	Severe	With more cardiac risk

The distinction between the different iron distribution patterns is an important aspect in the differential diagnosis of iron overload disorders; therefore, R2* of the spleen and the pancreas should also be reported and, if possible, evaluated as pathological or not [52].

**Fig. 6:**
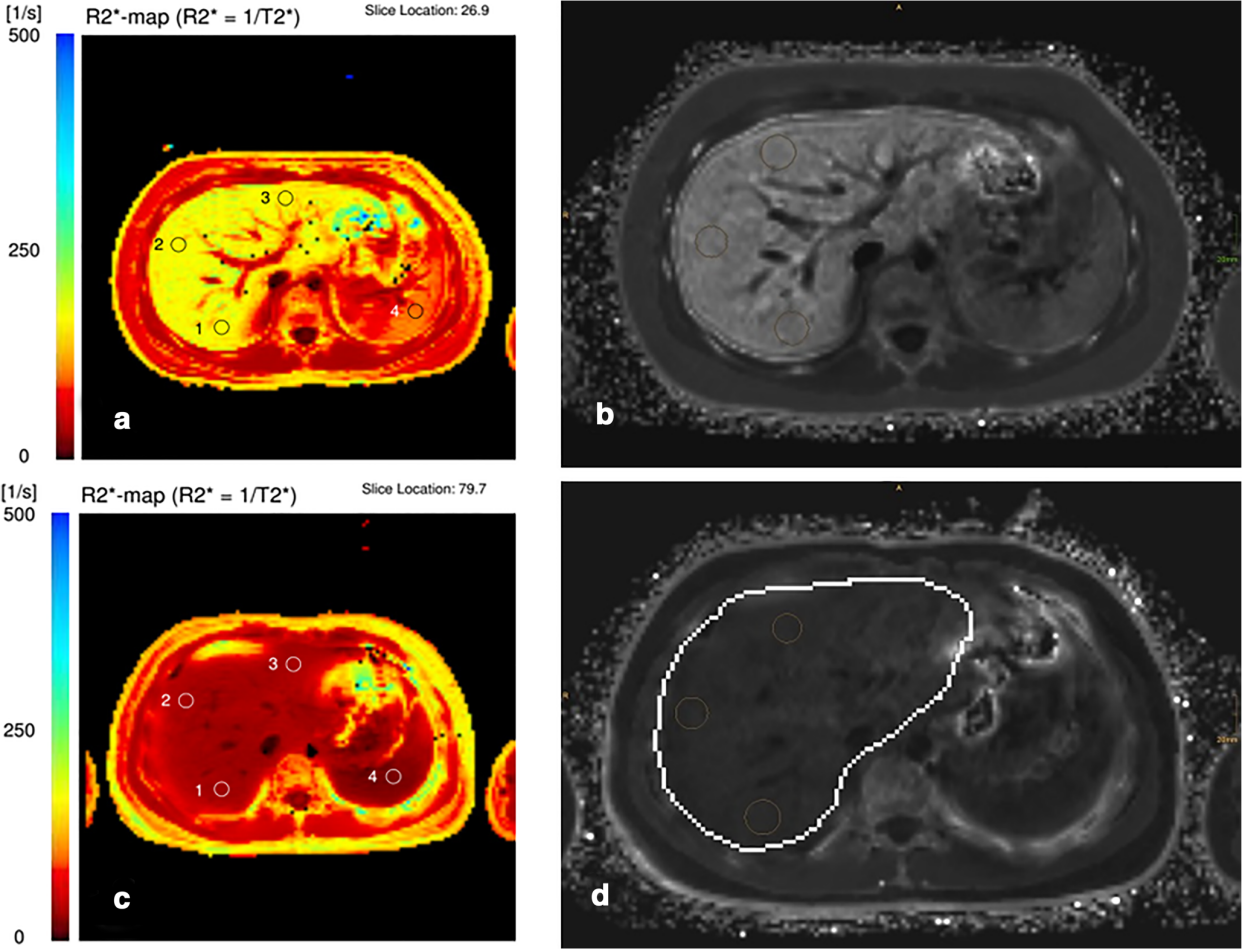
A 15-year old patient with secondary iron overload due to blood transfusion therapy. R2* with “in-house” ME-GRE sequences revealed a pathologic value of 162 s^−1^ (**a**), confirmed by qDixon (LiverLab) (**b**) with 169 s^−1^. After 2 years of chelation therapy, the values normalized to 35 s^−1^ (ME-GRE) (**c**)/32 s^−1^ (qDixon) (**d**). Further iron overload of the spleen was initially detected (R2* 102 s^−1^). Spleen values also decreased to a normal value under therapy (R2* 21 s^−1^)

In therapy monitoring, the baseline finding and the calculated LIC should be mentioned, if available. An example of monitoring a patient under chelation with MRI is provided in Fig. 6. We strongly suggested structured reporting. The software MRQuantif, proposed by the Rennes team, automatically builds a report and stores a data file.

## Closing comments

We are aware that in literature, concerning R2* relaxometry, there is strong doubt on its reproducibility with the fear that recalibration is necessary for any modifications on sequence parameters and post-processing. This “fear” is partly justified, of course, especially if you want to have a very accurate quantification. Measured R2* values depend an awful lot on how the images are processed. While these effects are relatively modest at LIC levels typically found in HH, they can become significant at higher LIC’s. It could be shown that changing sequence parameters and post-processing alters results but not to the expected extent [16–18].

The goal is to have a tool that can allow for different acquisition parameters, determine the best method of analysis, and provide an LIC value that is sufficiently reproducible whatever the brand of the device or even the magnetic field used. Until this type of software is scientifically validated, it is important that you choose your method, decide for the calibration formula that fits most to your settings, and stay with that method.

## Abbreviations

DIOS: Dysmetabolic iron overload syndrome
HH: Hereditary hemochromatosis
LIC: Liver iron concentration
ME-GRE: Multi-echo gradient-echo
MRI: Magnetic resonance imaging
SIR: Signal-intensity-ratio
TE: Echo time
